# Supplementary material for: Multi-Epitope DC Vaccines with Melanoma Antigens for Immunotherapy of Melanoma
Source: Vaccines (Basel). 2025 Mar 25;13(4):346. doi: 10.3390/vaccines13040346 (PMC12031154; doi:10.3390/vaccines13040346)
Supplement: Supplementary file 1 [file vaccines-13-00346-s001.zip › vaccines-3498193-supplementary.pdf]

vaccines

Supplementary Figures

Supplementary Figure S1

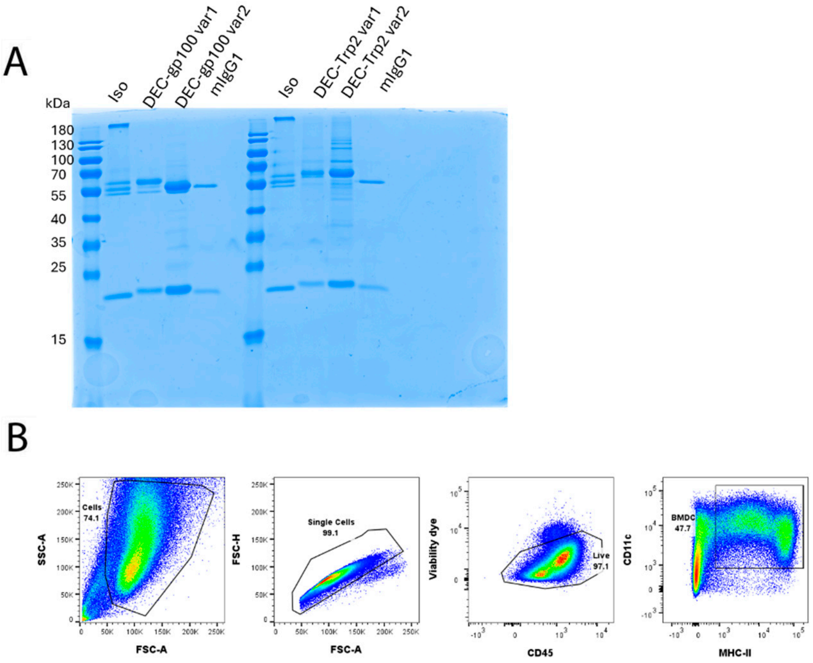

Supplementary Figure S1. Quality control of single epitope DC-vaccines. (**A**) SDS-Page gel showing the presence of heavy and light chains after antibody-antigen production for different DC-vaccines (**B**) Gating strategy for immature BMDC to verify binding of single-epitope DC-vaccines shown in Figure 1, representative for 3 experiments.

Supplementary Figure S2

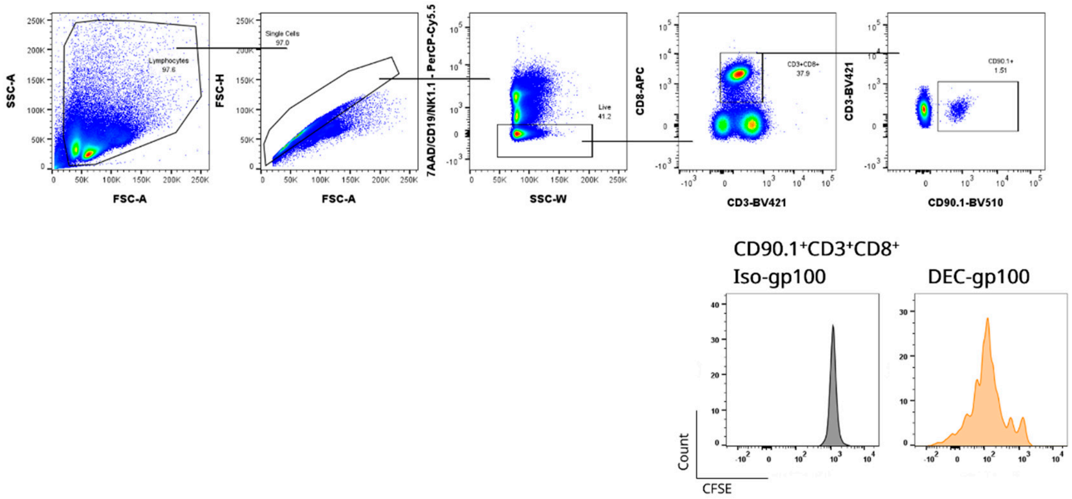

Supplementary Figure S2. Analysis of *in vivo* cross-presentation assays for DEC-gp100. Gating strategy for the *in vivo* CD8⁺ T cell proliferation assay shown in Figure 2. Transferred gp100-specific CD8+ T cells were gated of viable, lineage negative (non-B cells and non-NK cells) as CD3⁺CD8⁺ and congenic CD90.1⁺ marker. CFSE-dilution was used to determine proliferation of CD8⁺ T cells.

Supplementary Figure S3

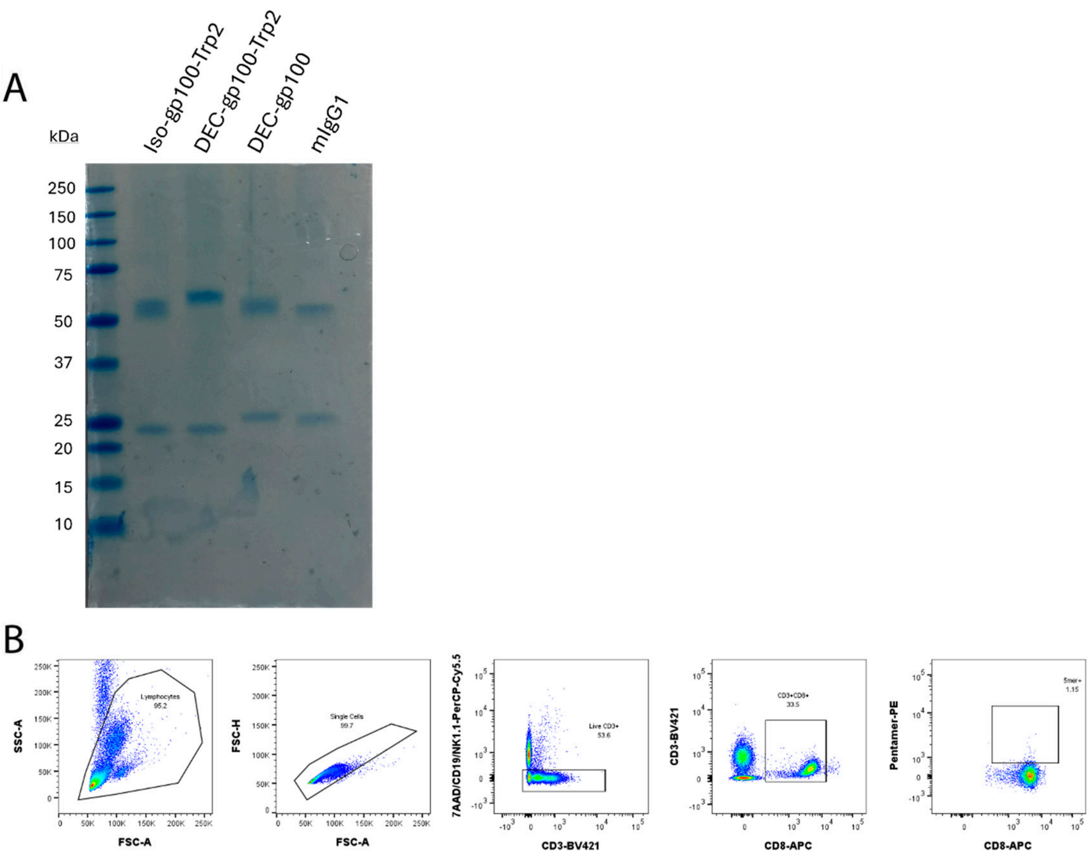

Supplementary Figure S3. Analysis of endogenous gp100- and trp2- specific CD8⁺ T cells after vaccination with DEC-gp100-Trp2 double-epitope DC-vaccine. Gating strategy for *in vivo* activated gp100- or trp2-specific CD8⁺ T cells from blood analyzed by flow cytometry. Pregate on viable (7AAD), lineage negative (CD19-NK1.1), CD90.1⁺, CD3⁺, CD8⁺.

Supplementary Figure S4

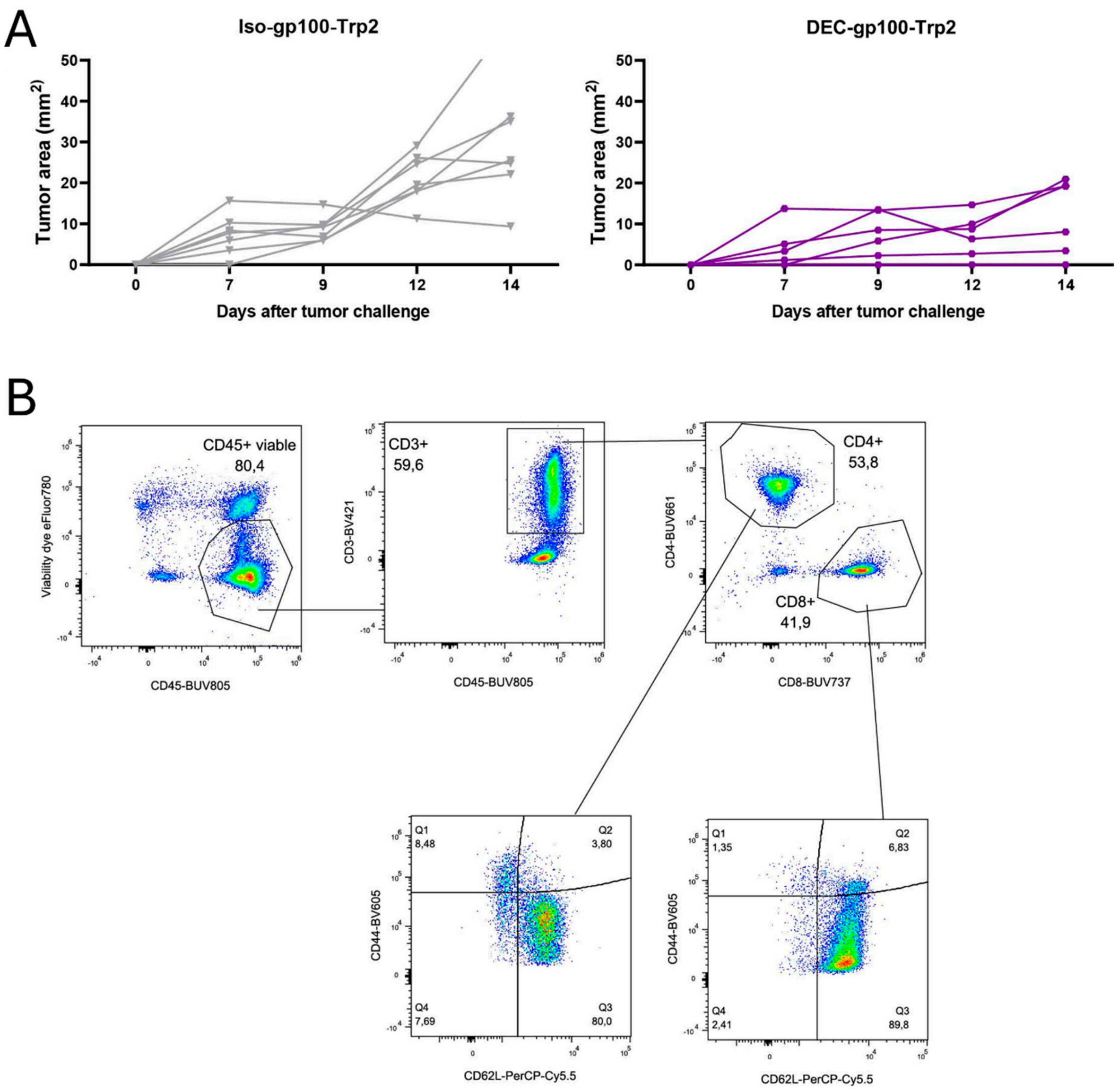

Supplementary Figure S4. Tumor growth curves and analysis of tumor-draining lymph nodes after treatment with DEC-gp100-Trp2. (**A**) Tumor growth curves after weekly treatment with DEC-gp100-Trp2 or Isotype-gp100-Trp2 control. Tumor experiments was stopped on day 14 to analyze tumor-draining lymph nodes. n=5-6 mice. (**B**) Gating strategy for T cells analyzed in tumor-draining lymph nodes on day 14 after tumor cell transfer.

Supplementary Figure S5

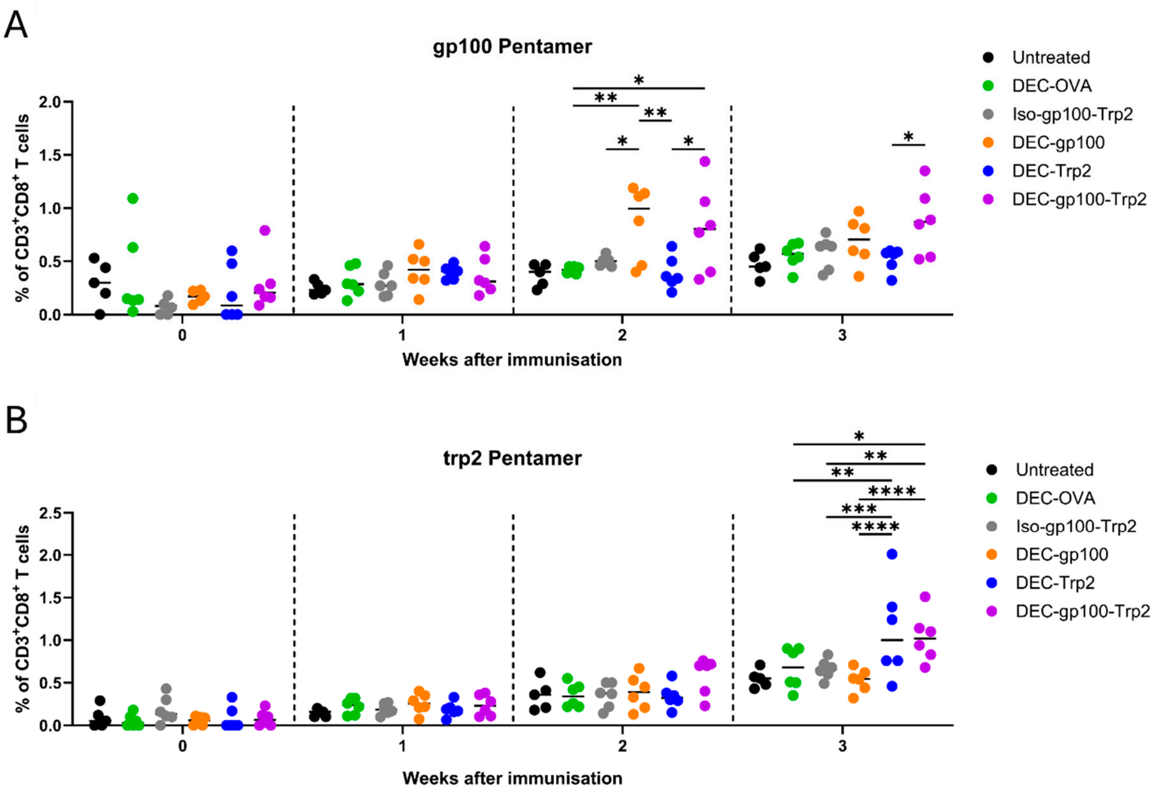

Supplementary Figure S5. Expansion of melanoma antigen-specific CD8+ T cells in tumor-bearing mice during treatment with DEC-gp100-Trp2. C57BL/6 mice were vaccinated subcutaneously into the flank skin with 5 µg of Iso-gp100-Trp2, DEC-OVA, DEC-gp100, DEC-Trp2 or DEC-gp100-Trp2 DC-vaccine in the presence of pI:C/aCD40 (12.5 µg each). Untreated controls received PBS. On day 9, the mice were challenged with 1.5 x10^5 B16.OVA cells by subcutaneous injection into the opposite flank skin. Blood samples were collected weekly on the day of vaccination and analysed by flow cytometry for gp100- (**A**) and trp2- (**B**) specific CD8+ T cells with pentamer stainings. Two-way ANOVA, Tukey multiple comparison test, n=5-6.
